# Ectomycorrhizal mediation of soil carbon sequestration: from carbon allocation to necromass stabilization and priming effects

**DOI:** 10.3389/fmicb.2026.1865735

**Published:** 2026-06-24

**Authors:** Yun-Xiao Han, Yong-Lian Wang, Mei-Hong Ge, Ye-Nan Wang, Dan Feng, Yang Yang, Dong-Ming Wu, Yu-Kun Zou

**Affiliations:** 1 Academy of Chemistry and Materials College, Hainan Vocational University of Science and Technology, Haikou, China; 2 National Agricultural Experimental Station for Agricultural Environment, Tropical Agro-ecosystem, National Observation, and Research Station, Danzhou, China; 3 Key Laboratory of Environmental Toxicology, Hainan University, Ministry of Education, Haikou, China; 4 Key Laboratory of Agro-Forestry Environmental Processes and Ecological Regulation of Hainan Province, Hainan University, Haikou, China; 5 Department of Resources and Environment, Moutai Institute, Renhuai, China; 6 Ministry Key Laboratory of Low-carbon Green Agriculture in Tropical region of China, Hainan Key Laboratory of Tropical Eco-circuling Agriculture, Institute of Environment and Plant Protection, Chinese Academy of Tropical Agricultural Sciences, Haikou, China

**Keywords:** ectomycorrhizal fungi, fungal necromass, nitrogen deposition, priming effect, soil carbon cycling

## Abstract

Ectomycorrhizal (ECM) fungi form the dominant symbiosis in many of the world's forest biomes. They exert a seemingly contradictory influence on soil carbon (C), simultaneously promoting C accrual through necromass inputs and aggregate protection, while also driving C loss via enzymatic priming. This review synthesizes current understanding of these dual roles, focusing on: (1) the magnitude and controls of photosynthetic C allocation from host plants to ECM mycelium; (2) the enzymatic mechanisms of SOM decomposition, the rhizosphere priming effect, and the contested universality of the “Gadgil effect” (competitive suppression of free-living saprotrophs); (3) physical and chemical stabilization pathways including the “microbial carbon pump” (necromass accrual) and “mineral carbon pump” (organo-mineral complexation); (4) environmental controls including nitrogen deposition, climate change, and forest management practices; and (5) prevailing research controversies and key methodological constraints—including isotope dilution, spatial heterogeneity of hyphal networks, and uncertain biomarker conversion factors—in quantifying fungal-mediated C fluxes. We identify key knowledge gaps, notably the need for explicit integration of ECM functional traits into ecosystem C models, resolution of the net balance between priming and stabilization under varying edaphic conditions, and a mechanistic understanding of how global change drivers alter ECM-C relationships. Future research should prioritize multi-scale approaches that integrate molecular omics, high-resolution isotope tracing, and process-based modeling to better constrain the role of ECM fungi in forest soil C sequestration and vulnerability. We further highlight that the ecological significance of ECM fungi in the global carbon cycle extends well beyond the well-studied northern forests, encompassing extensive tropical and Southern Hemisphere ECM systems that are subject to fundamentally different nutrient economies and global change pressures.

## 1 Introduction

Soils represent the largest terrestrial reservoir of organic carbon (C), storing an estimated 1,500–1,600 Pg C in the uppermost meter—a pool that exceeds the combined total of the atmosphere and global vegetation (Batjes, 1996; Jobbágy and Jackson, 2000). It should be noted that this global estimate includes peatlands and wetland soils, where anaerobic conditions fundamentally constrain decomposition through mechanisms distinct from those operating in the well-aerated mineral soils of ECM-dominated forests. The carbon cycling processes discussed in this review pertain primarily to the latter. Forest ecosystems account for approximately 60% of this belowground carbon, and a substantial portion of the world's forested area is dominated by tree species that form ECM associations (Read, 1991; Dixon et al., 1994). While the carbon-rich boreal and temperate forests of the Northern Hemisphere are classic examples of ECM dominance, the global footprint of this symbiosis extends far beyond high latitudes. ECM trees constitute the canopy dominants across vast tropical moist forests in Southeast Asia, the Miombo woodlands of Africa, and the ancient *Nothofagus* rainforests of the Southern Hemisphere, and form locally important stands in nutrient-impoverished white-sand forests of Amazonia (Corrales et al., 2018; Tedersoo et al., 2020). Collectively, these ECM-dominated biomes harbor immense soil carbon stocks but operate under fundamentally different climatic and edaphic constraints. Elucidating the mechanisms that govern both the stabilization and destabilization of soil C across this diverse geographic and environmental range is therefore essential for projecting ecosystem responses to global change and for informing forest management strategies aimed at enhancing C sinks (Jackson et al., 2017).

ECM fungi are central to this endeavor. In exchange for soil-derived nutrients—primarily nitrogen (N) and phosphorus (P)—these symbionts receive a substantial fraction of host photosynthates, with estimates ranging from 10% to 30% of net primary production (NPP; Hobbie, 2006; Ekblad et al., 2013). This continuous flux of recently fixed carbon from the canopy to the soil matrix via extensive extraradical mycelia constitutes one of the most significant, yet least constrained, conduits for C transfer from vegetation to soil in forest ecosystems (Clemmensen et al., 2013). However, the net consequences of this massive C allocation for long-term soil C storage remain both uncertain and a subject of active debate. This uncertainty stems directly from the multifaceted and seemingly contradictory roles that ECM fungi play once this carbon enters the belowground system (Averill et al., 2014; Fernandez and Kennedy, 2016).

From one perspective, ECM fungi are potent agents of carbon stabilization. Fungal biomass, extracellular secretions, and especially necromass (dead fungal residues) serve as direct inputs to soil organic matter (SOM), while the physical enmeshment of soil particles by extraradical hyphae promotes aggregate formation, thereby physically protecting organic matter from decomposition (Lehmann et al., 2017). Conversely, a contrasting body of evidence positions ECM fungi as drivers of carbon loss. Many ECM taxa retain an enzymatic repertoire capable of depolymerizing SOM, and their metabolic activity can accelerate the turnover of extant soil C through “priming” effects, potentially driving net C loss as they mine for organic nutrients (Talbot et al., 2008; Fernandez and Kennedy, 2016). This functional duality—the capacity to both stabilize and destabilize soil carbon—forms the central paradox in understanding ECM-mediated carbon cycling and is unlikely to be resolved without considering the strong environmental gradients across ECM-dominated biomes, particularly the shift from nitrogen limitation in high-latitude systems to pronounced phosphorus limitation in deeply weathered tropical soils (Allen et al., 2020; Almeida et al., 2023).

This inherent complexity is further compounded by ongoing debates surrounding specific mechanisms, such as the “Gadgil effect.” First described as the competitive inhibition of free-living saprotrophs by mycorrhizal fungi (Gadgil and Gadgil, 1975), its generality and underlying mechanisms have been vigorously contested. Recent investigations demonstrate that rather than universally suppressing decomposition, ECM fungi can accelerate SOM decomposition under specific contexts, challenging the universality of the Gadgil effect and highlighting the critical importance of context dependency in these interactions (Fernandez and Kennedy, 2016; Choreño-Parra and Treseder, 2024). These apparent discrepancies in the literature are not mere artifacts but reflect genuine context dependency driven by variation in fungal community composition, host tree identity, soil physicochemical properties, and prevailing environmental conditions. At the same time, they underscore persistent methodological challenges associated with quantifying carbon fluxes through ECM mycelia and distinguishing fungal-mediated transformations from those driven by other components of the soil microbiome.

**Figure 1:**
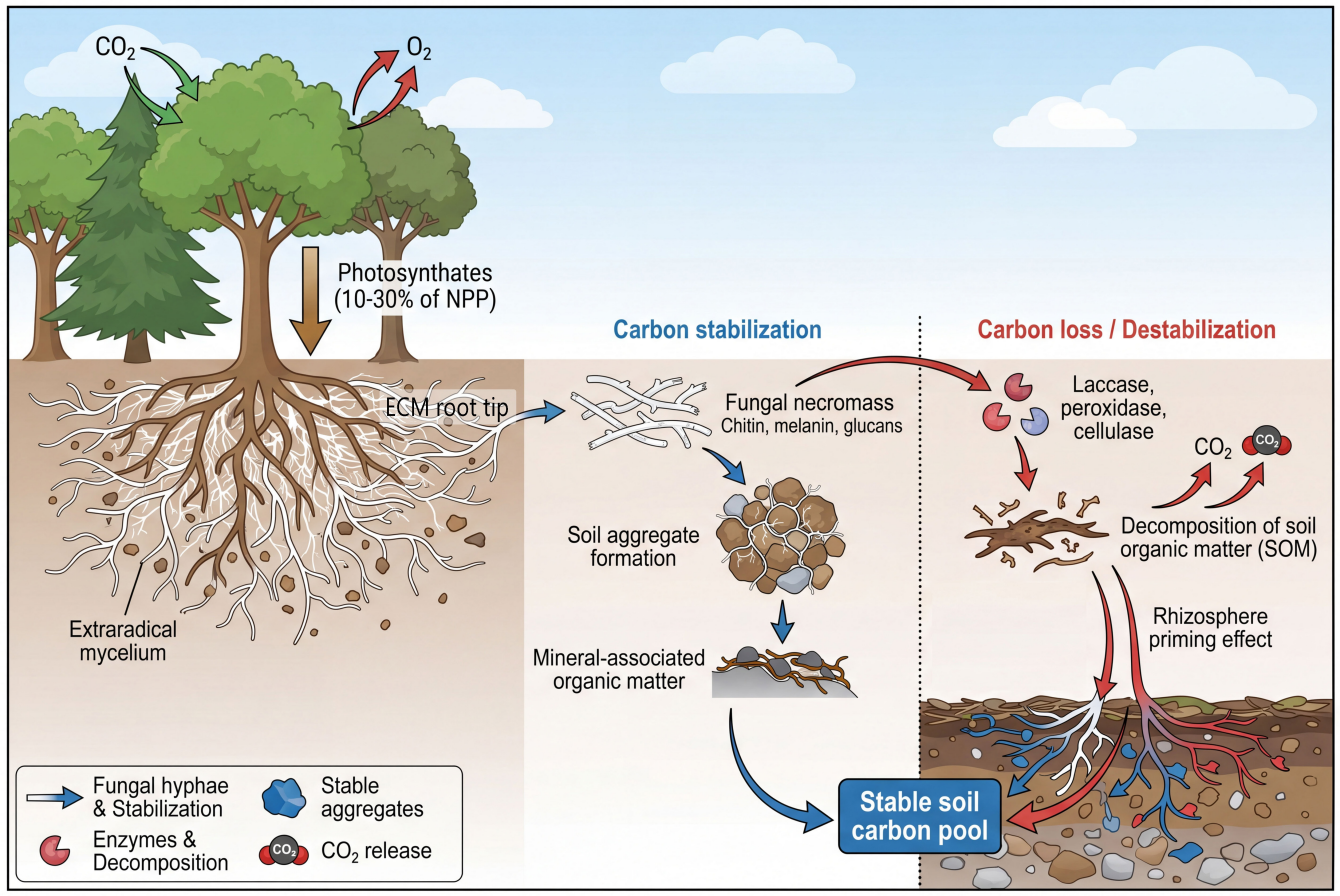
Graphical Abstract. Conceptual overview of ECM fungal roles in forest soil carbon cycling. Photosynthetic carbon (10–30% of NPP) allocated to ECM fungi enters two contrasting pathways: (i) stabilization via fungal necromass deposition, aggregate formation, and organo-mineral association (blue); and (ii) destabilization via enzymatic decomposition of soil organic matter and rhizosphere priming (red). The net outcome—whether ECM fungi function as a net carbon sink or source—depends on the balance between these processes, which is modulated by environmental conditions and fungal functional traits. This framework organizes the mechanisms examined in this review.

To navigate this complexity, this review aims to synthesize the current state of knowledge regarding ECM-mediated soil carbon cycling. This dualistic role—the simultaneous potential for C stabilization and loss—is conceptually synthesized in the graphical abstract (Figure 1), which provides the overarching organizing framework for the mechanisms examined in this review. We first examine the magnitude and regulation of plant C allocation to ECM symbionts, then dissect the contrasting mechanisms of SOM decomposition and stabilization, and finally evaluate the modulating influence of key environmental drivers, including nitrogen deposition, climate change, and forest management practices. By organizing these processes within a unified conceptual framework, we identify critical knowledge gaps and methodological constraints that limit our predictive understanding. We conclude by proposing priority directions for future research that prioritize multi-scale approaches—integrating molecular omics, high-resolution isotope tracing, and process-based modeling—to better constrain the net role of ECM fungi as either a carbon sink or source in the world's forests.

To reconcile these contrasting roles, it is useful to view ECM-mediated carbon fluxes through a mass-balance lens. Photosynthetic carbon allocated belowground is partitioned among four principal fates: (i) fungal respiration, (ii) the production of fungal biomass that can enter the necromass pool, (iii) the secretion of extracellular enzymes and metabolites that can fuel rhizosphere priming, and (iv) the physical and chemical stabilization of soil organic matter via hyphal enmeshment and mineral interactions. The net effect of ECM fungi on soil carbon storage thus depends on the relative proportion of carbon channeled into stabilization pathways vs. that released through catabolic and priming processes. This partitioning is not static: it is dynamically regulated by host nutrient demand, fungal community composition, and soil conditions—most notably nitrogen and phosphorus availability. Under severe nutrient limitation, a greater fraction of allocated carbon is directed toward enzyme production and SOM mining, tilting the balance toward carbon loss; when nutrients are more readily available, a larger proportion may be allocated to hyphal growth and necromass accrual, favoring stabilization. In the following sections, we first examine the magnitude and controls of photosynthetic C allocation to ECM fungi (Section 2), and then systematically dissect the stabilization mechanisms—the microbial carbon pump (Section 3) and the mineral carbon pump (Section 4)—before analyzing the enzymatic and priming-driven loss pathways (Section 5) and the environmental factors that modulate the balance between them (Section 6).

## 2 Photosynthetic carbon allocation effect of ECM fungi

As outlined in the conceptual framework above, quantifying the total carbon flux from host to fungus is the essential first step in constraining the overall carbon budget of ECM symbioses.

### 2.1 Magnitude of carbon flux

The allocation of photosynthetically fixed C to ECM fungi represents a major pathway of C flux from vegetation to soil. Estimates of the proportion of net primary production (NPP) allocated belowground to ECM fungi vary from < 1% to over 30%, depending on ecosystem type, soil fertility, and methodological approach. Across studies, the median allocation falls within approximately 10%−15% of NPP, with the broad range reflecting genuine biological variation as well as differences in whether allocation is expressed relative to NPP or gross primary production (GPP). In culture studies, (Hobbie 2006) reported a mean allocation of 11.3% of NPP (range 1%−21%), while field estimates typically range from 0% to 22% of NPP. A recent modeling study across temperate forests estimated ECM carbon costs at approximately 16% of NPP (Shao et al., 2025). Where values are reported relative to GPP, the proportional allocation is correspondingly lower (Litton et al., 2007; Ekblad et al., 2013; Allen and Kitajima, 2014). In boreal forests, where ECM associations dominate and soil N availability is often limiting, C allocation to ECM fungi may be particularly high. (Clemmensen et al. 2013) estimated that fungal necromass (primarily ECM) contributed approximately 70% of soil organic C accrual along a boreal forest chronosequence, suggesting massive C allocation to and turnover of fungal tissues. Whether similarly high contributions characterize tropical ECM forests remains an open question. In the highly weathered Ferralsols underlying many tropical ECM systems, phosphorus rather than nitrogen is the primary limiting nutrient. Under such conditions, the carbon cost of nutrient acquisition—and thus the fraction of net primary production allocated to ECM fungi—may differ fundamentally from boreal estimates, with potential consequences for the magnitude of fungal necromass inputs to soil carbon pools globally (Allen et al., 2020; Almeida et al., 2023). Similarly, (Ekblad et al. 2013) synthesized data from approximately 140 forest sites and estimated that extramatrical mycelium (EMM) production ranged from 20 to 980 kg C ha^−1^ yr^−1^, representing a substantial fraction of belowground C allocation. This nearly two-orders-of-magnitude variation is driven by latitude (boreal vs. temperate), stand age, soil fertility, and host tree species, with the highest values typically reported from young to mid-rotation boreal conifer stands (Ekblad et al., 2013). The majority of C allocated to ECM fungi is respired, with estimates suggesting 43–64% of allocated C is lost as CO_2_ through fungal respiration (Ek, 1997; Ekblad et al., 2013). The remaining C is incorporated into fungal biomass, secreted as exudates, or transferred to soil organic matter pools. However, these estimates are subject to considerable uncertainty due to methodological challenges in distinguishing fungal respiration from root and microbial respiration *in situ*.

### 2.2 Controls on carbon allocation

Carbon allocation to ECM fungi is regulated by both plant and fungal physiology, as well as environmental conditions. Plant carbon allocation to mycorrhizal fungi is often interpreted through the lens of optimal allocation theory, which posits that plants invest carbon preferentially in the nutrient-acquisition strategy that yields the highest return per unit of carbon expended (Johnson et al., 2013). Under this framework, theory predicts that plants should allocate more C to mycorrhizal partners when nutrient limitation is severe, and empirical studies generally support this prediction, however, the relationship between N availability and C allocation is complex and may depend on whether N limitation is relieved through mineral N availability or organic N mineralization (Corrêa et al., 2011; Hasselquist et al., 2016). P limitation can also drive C allocation to ECM fungi, though the relationship appears weaker than for N limitation in many systems, and in tropical ECM forests, P limitation may be more important than N limitation in driving mycorrhizal C costs (Allen et al., 2020; Almeida et al., 2023). Different tree species allocate different amounts of C to ECM fungi, potentially reflecting differences in mycorrhizal dependency, growth strategy, or tissue chemistry (Xie et al., 2024). Different ECM fungal species and functional types vary in their C demands and growth rates, with long-distance exploration types, which produce extensive extraradical mycelium, representing a larger C sink than contact or short-distance exploration types (Agerer, 2001; Hobbie and Agerer, 2010).

### 2.3 Tracking carbon allocation: methodological approaches

Quantifying C allocation to ECM fungi has proven challenging due to the intimate physical association between fungal hyphae and root tissues, and a fundamental difficulty in distinguishing ECM hyphae from those of saprotrophic fungi. Isotope tracing through pulse-labeling experiments with ^13^CO_2_ or ^14^CO_2_ allows tracking of photosynthetically fixed C into fungal biomass and respiration, and these studies typically find rapid transfer of labeled C from leaves to roots to ECM fungi, with peak labeling of fungal tissues occurring within hours to days (Johnson et al., 2002; Ekblad et al., 2013). Because the labeled carbon originates from the host plant, its detection in extraradical hyphae provides strong functional evidence for the ECM pathway, inherently distinguishing this flux from saprotrophic C uptake. Recent advances in high-resolution imaging have further refined this approach: coupling ^13^C-CO_2_ pulse labeling with nanoscale secondary ion mass spectrometry (NanoSIMS) now enables *in situ* visualization of newly assimilated carbon allocation from host roots to individual fungal hyphae at the micrometer scale, revealing the spatial heterogeneity of C transfer within the mycorrhizal interface and providing direct evidence for rapid hyphal transport of recent photosynthates into soil microsites (Kaiser et al., 2015; Vidal et al., 2021). The spatial proximity of labeled hyphae to the root cortex in these images offers unequivocal microscopic confirmation of the ECM origin. Ergosterol, a membrane sterol specific to fungi, and chitin, a cell wall component, can be used as biomarkers to estimate fungal biomass and its contribution to soil C, although conversion factors between these biomarkers and fungal C are uncertain and may vary with fungal species and tissue type (Ekblad et al., 1998). However, these bulk chemical markers cannot discriminate between ECM and saprotrophic fungi, and thus provide only an integrated measure of the total fungal pool. Phospholipid fatty acids (PLFAs) and neutral lipid fatty acids (NLFAs) can distinguish fungal from bacterial biomass and have been used to track C incorporation into fungal membranes (Olsson et al., 2005). However, a major limitation of PLFA/NLFA profiling alone is its inability to differentiate ectomycorrhizal (ECM) fungi from saprotrophic fungi, as many fatty acid biomarkers (e.g., 18:2ω6,9) are common to both guilds. To circumvent this, PLFA analysis can be coupled with stable isotope probing (SIP). In this approach, ^13^C-CO_2_ pulse labeling of host trees directs recently fixed photosynthates specifically to ECM symbionts. The subsequent detection of ^13^C enrichment in fungal-specific fatty acids (e.g., through gas chromatography–combustion–isotope ratio mass spectrometry, GC-C-IRMS) provides functional evidence that the labeled C was routed through the mycorrhizal pathway, effectively distinguishing ECM fungal C dynamics from those of free-living saprotrophs (e.g., Drigo et al., 2012; Olsson et al., 2005). This combined approach represents a powerful, though underutilized, tool for resolving ECM-specific C fluxes within the complex soil food web. DNA- and RNA-based methods can quantify fungal biomass and activity, while stable isotope probing (SIP) can track labeled C into specific fungal taxa (Drigo et al., 2012). When SIP is combined with high-throughput sequencing of taxonomic marker genes (e.g., ITS, 18S rDNA), it becomes possible to identify precisely which ECM fungal species have assimilated the labeled photosynthate, directly linking identity to function and conclusively separating ECM from non-ECM guilds within a complex community. Each of these methods has limitations, and estimates of C allocation vary depending on the approach used. Critically, the degree to which results can be specifically attributed to ECM fungi vs. the broader fungal community depends on whether the method inherently captures a mycorrhiza-specific signal (e.g., plant-derived ^13^C) or must be combined with additional discrimination techniques (e.g., SIP, imaging, or phylogenetic assignment). This methodological uncertainty contributes to ongoing debate about the quantitative importance of ECM fungi in forest C budgets. Table 1 provides a comparative summary of major methodological approaches for quantifying carbon allocation to ECM fungi, including their advantages and limitations.

**Table 1:** Methodological approaches for quantifying carbon allocation to ectomycorrhizal fungi.

Method category	Specific technique	Measured parameter	Advantages	Limitations
Isotope tracing	^13^C/^14^C pulse labeling	Dynamics of newly-assimilated carbon in hyphae	Real-time tracking of C flow; can be coupled with NanoSIMS for spatial resolution	Short-term labeling insufficient for long-term allocation patterns; rapid respiratory loss of the label can lead to underestimation of gross C allocation if only biomass incorporation is measured
Biomarker analysis	Ergosterol, chitin	Fungal biomass	Distinguishes fungi from bacteria	Conversion factors uncertain; cannot distinguish live vs. dead biomass
Fatty acid analysis	PLFA/NLFA (e.g., 18:2ω6,9)	Live fungal membrane lipids	High sensitivity; can distinguish taxonomic groups	Costly; some fatty acids not exclusive to fungi
Molecular methods	qPCR, ITS (internal transcribed spacer) amplicon, SIP	Fungal abundance and active taxa	Species-level resolution; SIP links function to taxa	Relative abundance only; DNA does not reflect metabolic activity
Field exclusion	Girdling, ingrowth cores	*In situ* hyphal production	Direct measurement under field conditions	Experimental disturbance; incomplete exclusion

PLFA, phospholipid fatty acid; NLFA, neutral lipid fatty acid; SIP, stable isotope probing; ITS, internal transcribed spacer.

## 3 Microbial carbon pump effect of ECM fungi

The fungal biomass produced from the allocated carbon described in Section 2 eventually enters the soil as necromass, constituting the first major stabilization pathway—termed the microbial carbon pump.

### 3.1 Fungal necromass as a carbon source

Fungal necromass (dead fungal residues) represents a significant input to SOM and may be particularly important for long-term C storage. (Liang et al. 2019) reported that model simulations suggest microbial necromass could contribute 47–80% (up to 82%) of SOC, while empirical data based on amino sugar biomarkers show lower contributions in forest soils (~30–35%). Fungal necromass dominates in forest soils (often >65% of total necromass). A global synthesis by (Wang et al. 2021) further supports this, showing that fungal necromass accounts for >65% of total microbial necromass, and that total microbial necromass contributes an average of 35% to forest soil organic carbon. It is important to distinguish ECM fungal necromass from that of other fungi, particularly arbuscular mycorrhizal fungi (AMF), whose residues differ substantially in chemical composition and turnover dynamics. AMF hyphae are relatively labile and decompose rapidly, whereas ECM necromass is often enriched in recalcitrant compounds and persists longer in soil. ECM fungal necromass has several characteristics that favor C persistence. Fungal cell walls contain chitin, glucans, and melanins, and the decomposition dynamics of these components differ fundamentally from those of plant-derived compounds (Fernandez and Koide, 2012; Mancinelli et al., 2023). While active fungal hyphae typically have a C:N ratio of 15–25, making them relatively labile substrates for microbial turnover, structural components of ECM necromass—particularly melanized cell walls and rhizomorphs—can exhibit significantly higher C:N ratios (up to 30–50 or more) and greater biochemical recalcitrance. Conversely, the more labile cytoplasmic fraction of necromass may become transiently nitrogen-enriched during early decomposition as colonizing microbes immobilize exogenous nitrogen, creating localized hotspots of microbial activity. The net persistence of ECM necromass thus depends on the relative proportion of recalcitrant structural vs. labile cytoplasmic components, and on the decomposer community's nitrogen economy during the early stages of decay (Brzostek et al., 2015; Beidler et al., 2025). This chemical heterogeneity means that while some fungal residues are rapidly cycled, the more recalcitrant fractions may contribute directly to the formation of stable organic-mineral complexes (see Section 4; Brzostek et al., 2015; note that this study compared tree mycorrhizal types rather than ECM fungal communities directly). Furthermore, fungal necromass is produced throughout the soil matrix, including within stable aggregates where it may be physically protected from decomposition. (Clemmensen et al. 2013) provided compelling evidence for the importance of fungal necromass in boreal forest soils, showing that soil C accumulation along a 120-year chronosequence was strongly correlated with the abundance of fungal-derived organic matter as indicated by amino sugar biomarkers. It should be noted that the majority of studies quantifying fungal necromass contributions to soil carbon have been conducted in nitrogen-limited boreal and temperate forests; the stoichiometry, chemical composition, and persistence of ECM necromass in phosphorus-limited tropical soils remain largely uncharacterized.

### 3.2 Amino sugar biomarkers

Amino sugar analysis has become a standard method for quantifying the contribution of microbial necromass to SOM. Glucosamine (GlcN) is a major component of fungal chitin and bacterial peptidoglycan, and fungal GlcN can be distinguished from bacterial GlcN based on the ratio of GlcN to muramic acid (MurA), which is found only in bacteria. In practice, a more reliable indicator is the ratio of fungal carbon to bacterial carbon (F/B) derived from amino sugar concentrations, where a value greater than 1 typically indicates fungal residue dominance (Joergensen, 2018). However, the interpretation of this metric is sensitive to soil conditions: evidence suggests the ratio can be influenced by soil properties such as pH, with lower pH generally favoring fungal residues (Li et al., 2022; Xia et al., 2019). Given this variability, site-specific calibration is therefore recommended rather than reliance on a single universal threshold. Muramic acid (MurA) is a unique component of bacterial cell walls and is used to estimate bacterial necromass contribution. Galactosamine (GalN) is less commonly used but can provide additional information about microbial community composition. Based on these biomarkers, (Liang et al. 2019) developed conversion factors to estimate fungal and bacterial contributions to SOC:


$$\begin{array}{l} \text{Fungal C }=\;\frac{\text{Glc}{\text{N}}_{\text{soil}}\times f_{\text{fungal}}}{k_{\text{fungal}}}\times C_{\text{conversion}} \end{array}$$

where *f*_fungal_ is the fraction of total soil glucosamine derived from fungi (calculated from the GlcN:MurA ratio), *k*_fungal_ is the average glucosamine content in fungal biomass (mg GlcN g^−1^ fungal biomass), and *C*_conversion_ is the carbon concentration of fungal biomass (mg C mg^−1^ fungal biomass). Based on the comprehensive synthesis of (Liang et al. 2019), recommended values are *k*_fungal_ = 49 mg GlcN g^−1^ fungal dry weight and *C*_conversion_ = 0.45 mg C mg^−1^ fungal dry weight, with *f*_fungal_ being sample-specific and derived from the GlcN:MurA ratio assuming a bacterial GlcN:MurA baseline of approximately 2:1.

Application of these methods has consistently shown higher fungal than bacterial contributions to SOM in forest soils (Wang et al., 2021).

## 4 Mineral (physical) carbon pump effect of ECM fungi

Beyond the biochemical stabilization of necromass, carbon allocated to ECM hyphae also promotes physical and chemical protection mechanisms “the mineral carbon pump” that further enhance the persistence of soil organic matter.

### 4.1 Soil aggregate formation and stabilization

ECM fungi promote soil aggregate formation through multiple mechanisms. Hyphae enmesh soil particles and smaller aggregates, forming larger structural units through physical binding, and ECM fungi secrete extracellular polysaccharides and other compounds that act as glues, binding soil particles together (Miller and Jastrow, 2000; Zheng et al., 2014; Rillig et al., 2015). Some ECM fungi also produce hydrophobic compounds that increase water repellency and aggregate stability (Zheng et al., 2014). Aggregate formation is important for C protection because aggregates create microenvironments where pore sizes are too small for many bacteria and fungi to access, extracellular enzymes may not diffuse effectively into aggregate interiors, limiting decomposition of encapsulated organic matter, and aggregate interiors may be anaerobic or microaerophilic, suppressing aerobic decomposition processes (Chenu and Stotzky, 2001; Allison, 2005; Akbari and Ghoshal, 2015; Naughton et al., 2021; Soto Gómez et al., 2023). While a meta-analysis by (Leifheit et al. 2014) demonstrated that arbuscular mycorrhizal fungi have an overall positive effect on soil aggregation, direct evidence for ECM fungi is provided by (Zheng et al. 2014), who showed that ECM inoculation of Pinus sylvestris seedlings significantly promoted macroaggregate formation and soil water repellency through hyphal enmeshment and hydrophobic compound production. Building on these findings, (Rillig et al. 2015) proposed a trait-based framework for understanding how plant roots and mycorrhizal fungi contribute to soil aggregation, identifying architectural traits such as hyphal density and physiological traits including water repellency as important parameters for predicting aggregate stabilization capacity.

### 4.2 Mineral interactions and organic-mineral complexes

In addition to aggregate protection, fungal-derived organic matter can be stabilized through direct interactions with soil minerals. This “chemical protection” mechanism involves organic compounds binding to mineral surfaces through ligand exchange reactions, forming stable organo-mineral complexes, divalent and trivalent cations (Ca^2+^, Fe^3+^, Al^3+^) bridging organic compounds to mineral surfaces, and sorption and surface complexation mediated by pH-dependent charge and polyvalent cations. (Kalbitz et al. 2005) demonstrated that fungal-derived organic compounds can form stable associations with soil minerals, contributing to long-term C storage. The importance of mineral interactions for fungal necromass stabilization may vary with soil type, being particularly important in high-activity clay soils (Beidler et al., 2025). Emerging evidence further elucidates the specific pathways through which ECM fungal necromass is incorporated into mineral-associated organic matter (MAOM). (Beidler et al. 2025) directly tracked the fate of fungal necromass carbon and nitrogen in temperate forest soils, revealing that a substantial portion was rapidly incorporated into the MAOM pool. This process was primarily mediated by abiotic sorption and microbial reworking, rather than physical entrapment alone, highlighting the synergistic role of soil minerals and the decomposer community in stabilizing fungal-derived C. In the same study, (Beidler et al. 2025) further demonstrated that the chemical composition of fungal necromass, particularly its melanin content, governs its stabilization efficiency on mineral surfaces. Non-melanized necromass exhibited higher sorption affinity and greater persistence on iron oxides compared to melanized necromass—likely because melanin coatings reduce the availability of carboxyl and phenolic hydroxyl functional groups that mediate ligand exchange with mineral surfaces—suggesting that the intrinsic biochemical traits of ECM fungi can directly influence the formation of stable organo-mineral complexes. Collectively, these studies underscore that the stabilization of ECM fungal residues is not merely a passive physical process but is actively controlled by the interactive effects of mineralogy, necromass chemistry, and microbial activity. These stabilization pathways have been predominantly investigated in soils derived from glaciated parent materials with high-activity clays; their operation in the kaolinite- and sesquioxide-dominated soils typical of tropical ECM forests warrants direct investigation.

## 5 Priming effect of ECM fungi

In contrast to the stabilization pathways detailed in Sections 3 and 4, a portion of the carbon allocated belowground fuels the production of extracellular enzymes and metabolites that can accelerate the decomposition of native SOM. This represents the loss side of the carbon balance, commonly described through rhizosphere priming and related mechanisms.

### 5.1 The decomposition paradox

A central paradox in understanding ECM-C relationships—termed the “decomposition paradox”—is that ECM fungi are mutualistic symbionts that obtain C primarily from their plant hosts, yet many possess enzymatic capabilities for decomposing SOM. This apparent contradiction has led to competing hypotheses about the net effect of ECM fungi on soil C storage. Traditional theory held that ECM fungi were primarily nutrient foragers, using limited enzymatic capabilities to access N and P from organic sources while relying on plant-derived C for their energy needs (Read, 1991). However, genomic and transcriptomic studies have revealed that many ECM fungi retain genes for decomposing various SOM components, including lignin, cellulose, and chitin (Floudas et al., 2012; Kohler et al., 2015). A large-scale comparative genomic analysis of 135 fungal genomes further showed that the transition from saprotrophy to symbiosis in ECM lineages involved widespread losses of plant cell wall-degrading enzymes acting on lignin and cellulose, while also co-opting ancestral genes for new symbiotic functions (Miyauchi et al., 2020).

### 5.2 Enzymatic capabilities

ECM fungi produce a range of extracellular enzymes that can depolymerize SOM. Laccases, peroxidases, and other oxidative enzymes can attack lignin and other recalcitrant compounds, and some ECM fungi, particularly those in the order Boletales, retain substantial lignin-degrading capabilities similar to their saprotrophic ancestors (Liers et al., 2011; Fujii et al., 2013). Comparative genomic analyses have confirmed that while ECM fungi have lost many of the plant cell wall-degrading enzymes present in their saprotrophic relatives, they have retained a reduced but functionally diverse set of such enzymes, and the degree of retention varies among ECM lineages (Miyauchi et al., 2020). Cellulases, hemicellulases, and pectinases allow access to plant cell wall carbohydrates, and many ECM fungi can decompose cellulose, though typically at lower rates than specialized saprotrophs (Cairney and Burke, 1994; Shah et al., 2015; Tunlid et al., 2022). Proteases, chitinases, and phosphatases target organic N and P sources, and these enzymes are central to the nutrient-foraging strategy of ECM fungi and may contribute to SOM decomposition even when C mineralization is not the primary objective. The expression of these enzymes varies among ECM species and in response to environmental conditions. For example, (Nicolás et al. 2019) demonstrated that the expression of SOM-degrading enzymes in *Paxillus involutus* is co-regulated by carbon and nitrogen availability, leading the authors to propose that the combined oxidative and hydrolytic activity is triggered by nitrogen limitation, thereby supporting the broader hypothesis that N scarcity drives ECM-mediated decomposition.

### 5.3 The Gadgil effect and its controversies

The Gadgil effect-suppressed litter decomposition in the presence of mycorrhizal roots-was first documented by (Gadgil and Gadgil 1975) in a laboratory study showing that decomposition of *Pinus radiata* litter was slower when mycorrhizal roots were present. The authors hypothesized that mycorrhizal fungi competed with saprotrophic decomposers for nutrients, thereby inhibiting saprotroph activity. This phenomenon has been widely cited and has influenced conceptual models of forest C cycling (Fernandez and Kennedy, 2016). However, recent research has challenged both the generality and the underlying mechanism of the Gadgil effect. Several studies have found no evidence of suppressed decomposition by ECM fungi, or even accelerated decomposition, and (Fernandez et al. 2020) found that the direction of ECM effects on decomposition depended on both litter type and ECM community composition (Lang et al., 2021; Carteron et al., 2022). While nutrient competition was the original explanation, other mechanisms may explain observed patterns, including direct antagonistic interactions, modification of litter chemistry by ECM fungi, or physical protection of litter by hyphal envelopment (Choreño-Parra and Treseder, 2024). Furthermore, many studies of the Gadgil effect have used trenching or girdling to exclude ECM fungi, treatments that also affect root exudation, soil moisture, and other factors that influence decomposition (Malik, 2019). A recent meta-analysis by (Choreño-Parra and Treseder 2024) found that mycorrhizal fungi overall had neutral to positive effects on decomposition, challenging the universality of the Gadgil effect. However, they also found significant heterogeneity among studies, with negative effects (Gadgil effects, i.e., suppression of free-living saprotrophs) occurring under certain conditions and positive effects (rhizosphere priming, i.e., direct enzymatic mining of SOM by ECM fungi) predominating under others.

It is important to distinguish the Gadgil effect from the rhizosphere priming discussed below: whereas the former represents a competitive ecological interaction that suppresses free-living saprotrophs, the latter reflects a physiological nutrient-acquisition strategy of the mycorrhizal symbiosis. Recognizing this mechanistic distinction helps explain why both phenomena can operate simultaneously within the same soil volume.

### 5.4 Rhizosphere priming effects

In contrast to the Gadgil effect, “rhizosphere priming” refers to enhanced SOM decomposition in the presence of roots and associated mycorrhizal fungi. This phenomenon has been extensively documented in agricultural systems (Kuzyakov, 2002; Cheng and Kuzyakov, 2005). A meta-analysis by (Huo et al. 2017) found that rhizosphere priming enhances soil organic matter mineralization by an average of 59% across all studies, with woody species producing the strongest effects. In ECM-dominated temperate forests, (Brzostek et al. 2015) showed that experimentally reducing belowground C supply via girdling decreased the activity of SOM-degrading enzymes by approximately 40%, indicating that ECM root-derived C substantially primes microbial decomposition. Despite these advances, quantitative studies that directly partition ECM fungal contributions from root-mediated priming in forest systems remain scarce. Proposed mechanisms for rhizosphere priming include root exudates and fungal secretions providing labile C that stimulates microbial activity, leading to co-metabolism of SOM; microbes decomposing N-rich SOM to meet their nutritional needs when N is limiting, driven by C inputs from roots and fungi; and root and hyphal growth disrupting soil aggregates, exposing previously protected SOM to decomposition (Dijkstra et al., 2006; Craine et al., 2007; Kuzyakov, 2010; Pausch et al., 2013; He et al., 2020). The balance between priming (enhanced decomposition) and stabilization (reduced decomposition) likely depends on environmental conditions, particularly N availability and the quality of SOM substrates. Under high-N conditions, ECM fungi may suppress decomposition by satisfying their N needs without extensive SOM mining, while under low-N conditions, SOM decomposition may be enhanced to acquire N. This conceptual model has been developed primarily from studies conducted under nitrogen-limited conditions. Whether an analogous “phosphorus priming” mechanism operates in P-limited ECM systems, and whether its carbon cost is comparable to that of nitrogen-driven priming, remains an open question.

Table 2 summarizes the positive and negative effects of ECM fungi on soil carbon cycling, contrasting stabilization mechanisms (necromass input, aggregation, mineral association) with destabilization pathways (priming, enzymatic decomposition, and the controversial Gadgil effect).

**Table 2:** Positive and negative effects of ectomycorrhizal fungi on soil carbon cycling.

Direction	Primary mechanism	Mode of action	Key evidence
Carbon stabilization	Fungal necromass input	Chitin, melanin, and other recalcitrant compounds directly contribute to soil organic carbon	Clemmensen et al., 2013; Wang et al., 2021
	Soil aggregate formation	Hyphal entanglement and exudate binding physically protect carbon from decomposition	Rillig et al., 2015; Zheng et al., 2014
	Mineral-associated organic matter stabilization	Fungal residues sorb to mineral surfaces (e.g., Fe/Al oxides) forming stable organo-mineral complexes	Beidler et al., 2025
	Indirect Gadgil effect	Competitive suppression of free-living saprotrophs slows native SOM decomposition (an ecological interaction; distinct from active C stabilization by hyphae or necromass)	Gadgil and Gadgil, 1975; Fernandez and Kennedy, 2016; Choreño-Parra and Treseder, 2024
Carbon loss	Rhizosphere priming effect	Enzymatic decomposition of native SOM to acquire nitrogen, releasing CO_2_	Fernandez and Kennedy, 2016; Talbot et al., 2008
	Enzymatic decomposition	Production of laccases, peroxidases, cellulases directly depolymerizing SOM	Kohler et al., 2015; Nicolás et al., 2019

SOM, soil organic matter; ECM fungi can exert both positive (stabilization) and negative (loss) effects on soil C pools simultaneously. The Gadgil effect is classified here as a stabilization mechanism because it suppresses decomposition, but it is an indirect ecological effect—competitive inhibition of saprotrophs—distinct from rhizosphere priming, which is a direct physiological strategy of nutrient acquisition via enzymatic SOM mining.

**Figure 2:**
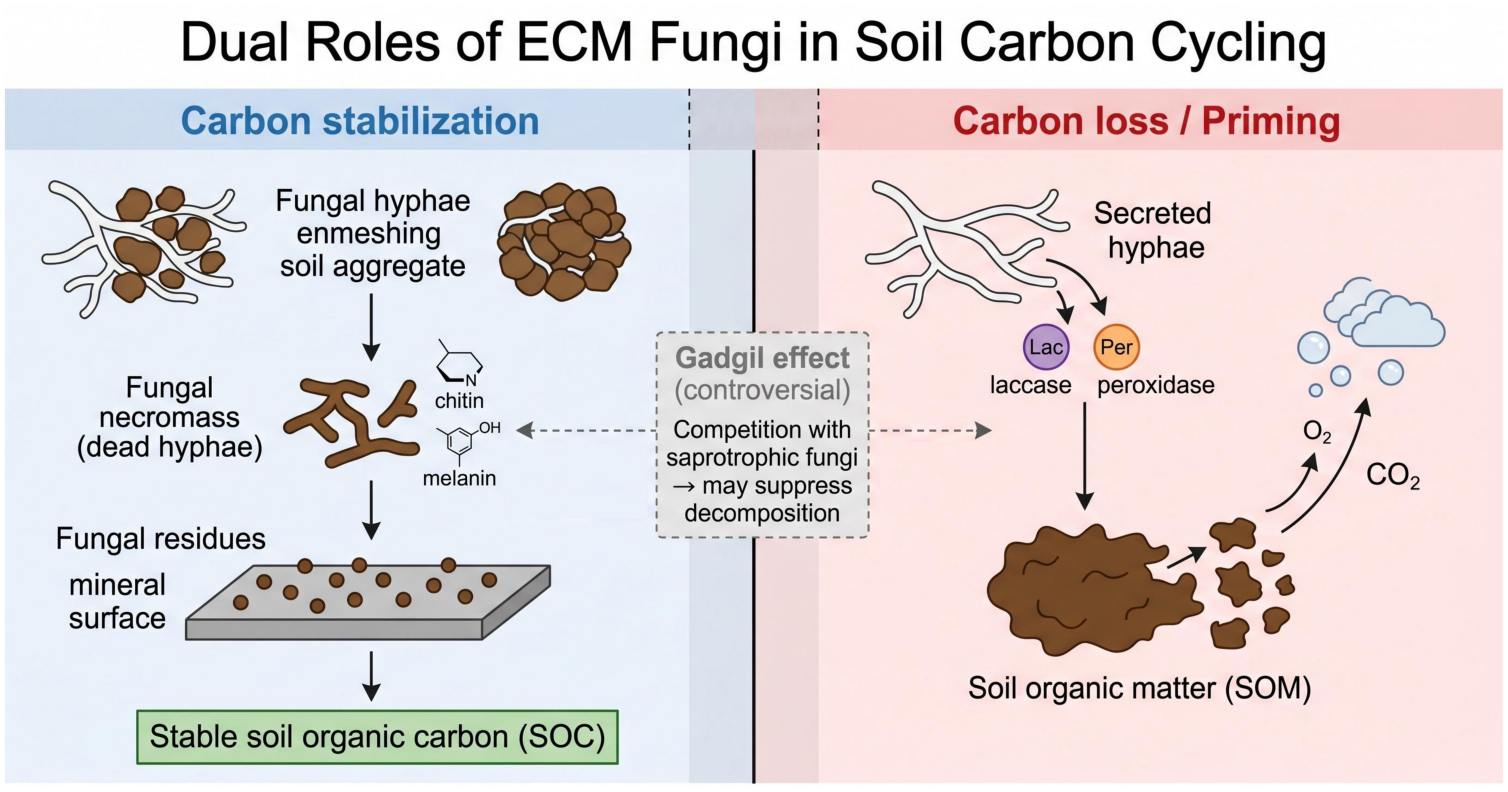
Mechanisms of ECM-mediated soil carbon stabilization and loss. The left panel depicts stabilization pathways: (i) physical protection through hyphal enmeshment and macroaggregate formation, and (ii) chemical protection via sorption of fungal necromass onto mineral surfaces (formation of mineral-associated organic matter, MAOM). The right panel depicts carbon loss pathways: (i) direct enzymatic mining of SOM by ECM fungi (oxidative and hydrolytic enzyme secretion) to acquire nutrients, resulting in CO_2_ release, and (ii) rhizosphere priming, whereby labile fungal exudates stimulate microbial activity and accelerate SOM turnover. Positioned between these two panels, the Gadgil effect (competitive suppression of free-living saprotrophs) is depicted as an indirect modulator that slows decomposition (stabilization side) yet is mechanistically distinct from and trades off with enzymatic priming (loss side). While both stabilization and priming operate simultaneously, their net effect on soil carbon storage is contingent upon edaphic conditions (e.g., nitrogen vs. phosphorus limitation) and ECM community composition.

The contrasting pathways of stabilization and loss, and their dependence on edaphic conditions and fungal community composition, are summarized in Figure 2.

## 6 Effects of environmental factors on carbon sequestration by ECM fungi

The relative dominance of stabilization vs. loss pathways is not fixed but is strongly modulated by environmental conditions. The following section examines how key global change drivers—including nitrogen deposition, climate change, and land management—shift the balance between carbon accrual and carbon release in ECM-dominated forests.

### 6.1 Nitrogen deposition

Atmospheric N deposition, a major component of global change, has profound effects on ECM fungi and their role in C cycling. Yet the nature and magnitude of global change pressures are not uniform across the distribution of ECM forests. While northern biomes are predominantly shaped by nitrogen deposition and rapid warming, tropical ECM forests are increasingly threatened by deforestation, land-use conversion, and drought intensification linked to large-scale climate modes (e.g., El Niño–Southern Oscillation, Indian Ocean Dipole). N addition generally reduces ECM fungal biomass, diversity, and activity, with consequences for soil C dynamics (Van der Linde et al., 2018; Cox, 2026). N deposition reduces plant C allocation to ECM fungi, thereby decreasing fungal biomass and necromass inputs (Janssens et al., 2010; Högberg et al., 2021). It also shifts ECM communities toward less mutualistic species with lower C demands (Weese et al., 2015; Lilleskov et al., 2019; Carrara et al., 2023). The net effect on decomposition is contradictory: N addition can either enhance decomposition by alleviating N limitation or suppress it by reducing microbial biomass (Liu et al., 2023). (Averill et al. 2018) found that N deposition was associated with reduced soil C stocks in AM-dominated but not ECM-dominated forests, suggesting that mycorrhizal type mediates ecosystem responses to N deposition.

### 6.2 Climate change

Climate warming and altered precipitation regimes affect ECM fungi and soil C cycling through multiple pathways. Warming generally increases soil respiration and may accelerate decomposition of SOM, and ECM fungal respiration is also temperature-dependent, potentially altering the balance between C allocation to fungi and C storage in soil (Malcolm et al., 2008). Reduced soil moisture can suppress fungal activity and C allocation, and (Querejeta et al. 2021) found that reduced precipitation favored drought-tolerant ECM species but decreased overall ECM abundance, potentially reducing fungal contributions to soil C storage. Rising atmospheric CO_2_ may increase overall plant productivity, as evidenced by a conserved 23% stimulation of forest NPP, potentially increasing C allocation belowground. However, the magnitude and persistence of this effect on soil C inputs remain uncertain (Norby et al., 2005).

### 6.3 Forest management

Forest management practices alter ECM communities and soil C dynamics. Removal of host trees through clearcutting eliminates the C supply to ECM fungi, leading to rapid declines in ECM mycelial biomass and potential losses of fungal-derived soil C (Jones et al., 2003; Kyaschenko et al., 2017; Parladé et al., 2017; Hasby, 2022). Selective harvest through thinning reduces C allocation belowground but maintains some host trees, potentially allowing ECM communities to persist at reduced biomass. N or P fertilization generally reduces ECM fungal biomass and activity, with potential negative effects on soil C storage (Fernandez and Kennedy, 2026). Plantation forestry with ECM tree species maintains ECM fungi and their contributions to soil C, though monocultures may support less diverse fungal communities than natural forests (Goldmann et al., 2015; Averill and Hawkes, 2016; Werner et al., 2024).

Table 3 summarizes how key environmental factors-including nitrogen deposition, climate warming, drought, elevated CO_2_, clearcutting, and fertilization-influence ECM fungi and their mediated soil carbon dynamics. Understanding these environmental controls is essential for predicting forest soil C responses to global change.

**Table 3:** Summary of environmental controls on ectomycorrhizal fungi–carbon relationships.

Environmental factor	Trend	Effect on ECM fungi	Potential effect on soil carbon pool	Key literature
Nitrogen deposition	Increasing	Decreased biomass; community shifts toward low-C-demand species	Reduced necromass input, potentially decreasing C sequestration	Carrara et al., 2023; Averill et al., 2018
Climate warming	Temperature increase	Increased respiration; extended growing season but may exacerbate drought stress	Potential increase in annual C allocation to ECM fungi from an extended growing season, which may be partially or fully offset by increased respiratory losses; long-term net effect uncertain	Malcolm et al., 2008
Drought	Decreasing precipitation	Reduced overall abundance; increased proportion of drought-tolerant species	Decreased contribution of hyphal turnover to C pool	Querejeta et al., 2021
Elevated CO_2_	Increasing	Enhanced belowground C allocation; increased hyphal growth	Potentially increased fungal necromass C input	Norby et al., 2005
Clearcutting	Host removal	Rapid hyphal death; loss of ECM function	Short-term release of fungal-derived C; long-term reduced input	Parladé et al., 2017; Jones et al., 2003
Fertilization (NPK)	Nutrient enrichment	Reduced mycorrhizal dependency; lower hyphal biomass	Reduced potential for soil C sequestration	Fernandez and Kennedy, 2026

Thus, the strong modulation by environmental factors is a key reason for the conflicting conclusions regarding stabilization vs. priming effects across different studies. This context directly highlights the core controversies and knowledge gaps that urgently need to be addressed in the current research field.

### 6.4 Tropical land-use change and southern hemisphere climate forcings

The ecological significance of ECM fungi in global carbon cycling is amplified when considering ongoing transformations in tropical and Southern Hemisphere ECM forests. In Southeast Asia, extensive conversion of ECM-dominated dipterocarp forests to oil palm or *Acacia* plantations represents a large-scale functional shift in mycorrhizal dominance—from ECM to arbuscular mycorrhizal (AM) systems. Emerging evidence suggests that this conversion reduces inputs of recalcitrant fungal necromass and diminishes the formation of mineral-associated organic matter, potentially eroding the stable soil carbon fraction characteristic of intact ECM forests (e.g., Van Straaten et al., 2015). In the Southern Hemisphere, ECM-dominated *Nothofagus* forests of Patagonia and New Zealand are experiencing increased drought stress linked to positive phases of the Southern Annular Mode (SAM). Unlike the permafrost-thaw feedbacks of the Arctic, the vulnerability of these ancient southern ECM systems lies in the synergistic effects of reduced hyphal exploration, increased wildfire risk, and the potential for rapid, non-linear carbon loss from organic-rich soils (Querejeta et al., 2021). Australian eucalypt ECM forests face similar pressures from intensifying drought and fire regimes. Collectively, these regionally distinct pressures underscore that ECM fungi are not merely mediators of northern carbon sinks but are globally distributed regulators of terrestrial carbon–climate feedbacks under divergent global change trajectories. The contrasting nutrient economies of tropical vs. high-latitude ECM forests raise a more fundamental question: how do ECM-mediated carbon cycling mechanisms differ under nitrogen vs. phosphorus limitation? We address this question in the following section.

### 6.5 ECM carbon cycling under nitrogen vs. phosphorus limitation: a contrasting framework

Our mechanistic understanding of ECM carbon cycling is largely derived from nitrogen-limited boreal and temperate forests. Yet extensive ECM-dominated biomes exist on phosphorus-impoverished tropical soils, representing fundamentally different biogeochemical challenges that likely drive divergent fungal strategies. Under nitrogen limitation, ECM fungi invest in extracellular oxidative and hydrolytic enzymes to mine organic nitrogen, incurring a high carbon cost through respiratory losses and priming-induced SOM mineralization. Under phosphorus limitation, the economics are less clear, but emerging evidence suggests a shift toward phosphatase secretion and organic acid exudation to desorb mineral-bound phosphate, a strategy that may involve less direct SOM oxidation and lower priming intensity (Allen et al., 2020; Almeida et al., 2023).

These contrasting nutrient economies also influence necromass chemistry and stabilization. Nitrogen-limited systems produce chitin- and melanin-rich necromass with moderate C:N ratios that facilitate mineral-associated organic matter (MAOM) formation. Phosphorus limitation is expected to elevate fungal C:P ratios and melanization, potentially increasing biochemical recalcitrance. However, (Beidler et al. 2025) demonstrated that non-melanized necromass sorbs more strongly to iron oxides, raising the paradox that P-limited necromass may be more recalcitrant yet less efficiently stabilized on the sesquioxide-rich minerals typical of weathered tropical soils. This interplay between fungal stoichiometry, melanization, and mineralogy remains unexplored in tropical ECM systems.

The distinct enzymatic strategies carry contrasting implications for SOM stability. The “nitrogen mining” paradigm, driven by peroxidases and laccases, incidentally mineralizes substantial carbon (Nicolás et al., 2019). A “phosphorus mining” strategy, dominated by phosphatases targeting specific ester bonds, is predicted to cause less collateral carbon loss. This prediction is complicated by stoichiometric theory, as severe phosphorus scarcity can induce microbial nitrogen immobilization and compensatory N mining (Craine et al., 2007). The net balance between these processes, and their response to environmental drivers, remains empirically unresolved.

We propose a working hypothesis: nitrogen limitation drives heavy investment in oxidative enzymes, substantial SOM oxidation, and the production of necromass whose stabilization relies on aggregate occlusion and mineral sorption. Phosphorus limitation favors phosphatase-dominated strategies with lower direct SOM oxidation but produces high C:P, melanized necromass whose long-term fate is strongly modulated by the sesquioxide-rich mineral matrix of tropical soils. Testing this hypothesis requires coordinated cross-biome studies that quantify carbon allocation, enzyme profiles, necromass chemistry, and stabilization dynamics under field conditions across both high-latitude and tropical ECM forests. Only through such comparative efforts can the role of ECM fungi in soil carbon cycling be understood with genuine global generality.

## 7 Research controversies and knowledge gaps

### 7.1 Methodological uncertainties in quantifying ECM-mediated carbon fluxes

A major source of the persistent uncertainty surrounding the net carbon balance of ECM symbioses lies in the methodological challenges inherent to measuring fungal-mediated carbon fluxes in complex soil environments. While Table 1 summarizes individual method limitations, the cumulative propagation of these errors through carbon budget calculations warrants systematic examination. Here we assess three interconnected methodological bottlenecks that underlie many of the contradictory conclusions in the current literature.

#### 7.1.1 Distinguishing fungal respiration from root and bulk microbial respiration

Partitioning soil CO_2_ efflux among root respiration, ECM fungal respiration, and free-living microbial respiration remains a fundamental challenge (Ekblad et al., 2013). Trenching or girdling treatments used to isolate fungal contributions simultaneously alter root exudation, soil moisture, and nutrient availability, introducing artifacts that may rival the fluxes being measured (Malik, 2019). Fungal respiration is often calculated as a residual term, compounding errors from all other flux components. Compound-specific isotope analysis and position-specific ^13^C labeling have begun to address this problem by distinguishing respiratory CO_2_ from different metabolic pathways (Dippold et al., 2015), but these approaches have yet to be applied systematically to ECM field studies. Without reliable respiratory partitioning, gross carbon allocation to ECM fungi cannot be constrained with confidence.

#### 7.1.2 Uncertainties in biomarker conversion factors for necromass estimation

Amino sugar biomarkers are the primary tool for quantifying microbial necromass contributions to SOC (Liang et al., 2019), yet their translation into absolute carbon masses requires conversion factors that remain a significant source of uncertainty. The GlcN:MurA ratio used to calculate the fungal glucosamine fraction varies among bacterial taxa and soil types, and the glucosamine content per unit fungal biomass (k_fungal) and carbon concentration (C_conversion) differ among ECM species, tissue types, and degradation states. Empirical evidence demonstrating that fungal necromass can account for 30–80% of SOC—depending on ecosystem and conversion assumptions—illustrates the wide margins of uncertainty introduced at this single step alone (see Section 3). These uncertainties are compounded when amino sugar data parameterize ecosystem models sensitive to necromass input rates and residence times.

#### 7.1.3 Capturing stabilization and priming simultaneously across scales

The net effect of ECM fungi on soil carbon depends on the dynamic balance between simultaneous stabilization and loss processes, yet most studies are designed to quantify one or the other, not both. This separation introduces a fundamental scale mismatch: stabilization operates over decadal to centennial timescales at pedon to landscape scales, whereas priming is typically measured in short-term incubations (hours to weeks) at aggregate or rhizobox scales. Scaling incubation-derived priming rates to ecosystem-level budgets requires untested assumptions about temporal persistence and spatial extent. Furthermore, fractionation techniques that separate mineral-associated organic matter physically disrupt the hyphal networks and aggregate architecture that mediate the processes under investigation. Emerging tools—continuous ^13^C labeling in free-air enrichment experiments, high-frequency automated soil respiration chambers, and non-destructive imaging (e.g., X-ray computed tomography)—offer pathways toward integrated frameworks that simultaneously track carbon inputs, mineralization, and physical protection over ecologically relevant scales.

Collectively, these methodological uncertainties directly propagate into divergent estimates of whether ECM fungi act as a net carbon sink or source. Recognizing and quantifying their magnitude is essential for designing experiments capable of resolving the current impasse and for developing models that appropriately represent prediction error envelopes.

### 7.2 Net effect and modeling challenges

Despite considerable research, the net balance between ECM-mediated stabilization (necromass accrual, aggregation) and destabilization (priming, enzymatic decay) remains unresolved (Averill et al., 2014; Choreño-Parra and Treseder, 2024). This uncertainty is compounded by a scale mismatch: incubation experiments typically last weeks to months, whereas soil carbon turnover operates over decades to centuries; molecular tracing (e.g., NanoSIMS) resolves processes at the micrometer scale, while ecosystem model grid cells span kilometers. Bridging these scales requires integrating fine-scale mechanistic data into process-based models that operate at management-relevant resolutions.

A major impediment to resolving this net balance is the lack of explicit ECM representation in ecosystem models (Wieder et al., 2014). Recent trait-based frameworks have begun to address this by incorporating ECM functional diversity, linking nitrogen acquisition strategies directly to carbon use efficiency (Jörgensen et al., 2025). Integrating such models with dynamic enzyme production—switching between stabilization and priming modes in response to nutrient status—is essential for improving projections of forest soil carbon vulnerability (Brzostek et al., 2014; Sulman et al., 2017).

A concrete illustration is provided by the MIMICS+ model (Aas et al., 2024), which explicitly distinguishes ECM from AM functional groups and represents microbial CUE as a function of substrate stoichiometry. We identify three priority trait groups requiring parameterization: (i) Exploration-type-dependent C allocation and hyphal turnover. Exploration types differ markedly in hyphal biomass, extension rate, and lifespan (Agerer, 2001). These can be parameterized as type-specific CUE, turnover rate, and biomass-to-necromass conversion efficiency, constrained by observed EMM standing biomass (20–980 kg C ha^−1^; Ekblad et al., 2013). (ii) Enzyme spectra linked to nutrient limitation. Rather than a single generic enzyme pool, models should represent oxidative (laccase, peroxidase) and hydrolytic (protease, phosphatase) enzyme classes with distinct SOM mineralization efficiencies. Allocation to oxidative enzymes could be parameterized as a function of soil C:N ratio, informed by metatranscriptomic data (Nicolás et al., 2019). (iii) Necromass chemistry and mineral sorption. The partitioning of necromass into mineral-associated organic matter depends on melanin content and soil mineralogy (Beidler et al., 2025), parameters that can be bounded using isolates with known melanization phenotypes and soil-specific reactive mineral surface areas.

Sensitivity analyses with MIMICS+ identify the most influential uncertainties as ECM CUE, hyphal turnover rate, and enzyme kinetic parameters (Vmax, Km). Critically, the net model outcome—sink vs. source—can reverse when CUE is varied within its empirically observed range (0.25–0.45; Jörgensen et al., 2025), underscoring the need for trait-specific rather than guild-averaged parameterization. We advocate a coordinated strategy of community-level trait surveys along nutrient gradients, continuous ^13^C labeling for simultaneous flux validation, and iterative benchmarking against data from understudied tropical and Southern Hemisphere ECM systems.

### 7.3 Scaling and methodological frontiers

Scaling understanding from hyphal tips to ecosystems is confounded by spatial heterogeneity and temporal dynamics that are rarely captured in manipulative experiments. Bridging this gap requires methodological innovation. Advances in compound-specific and position-specific isotope labeling provide finer resolution of carbon fluxes into distinct biochemical pathways (Dippold et al., 2015). Meanwhile, high-resolution imaging (e.g., NanoSIMS) reveals the spatial coupling between fungal hyphae and mineral surfaces at the microscale (Vidal et al., 2021), and metatranscriptomics increasingly links specific fungal taxa to the active decomposition of soil organic matter (Nicolás et al., 2019; Law et al., 2022). These tools are critical for moving beyond correlative biomass estimates to a mechanistic understanding of carbon fate.

## 8 Future perspectives and research priorities

### 8.1 Integrative research approaches

Future research on ECM-mediated C cycling should embrace integrative approaches that combine multi-scale studies linking molecular-level understanding of fungal physiology with plot-level C flux measurements and ecosystem-scale modeling. Establishing long-term experimental platforms to track ECM-C relationships over time scales relevant for C storage (decades to centuries) is also necessary, along with coordinated research across climate gradients through global networks to understand how ECM-C relationships vary with environmental conditions.

### 8.2 Application to forest management and climate mitigation

Understanding ECM-C relationships has direct applications for forest management. Choosing ECM tree species or provenances that maximize C allocation to stable fungal necromass could enhance soil C sequestration. Managing harvest intensity and timing to minimize disruption of ECM networks and maintain fungal contributions to soil C is another important silvicultural practice. Inoculating degraded soils with ECM fungi to restore C cycling functions and promote soil C accumulation may also be beneficial, though practical application faces obstacles including low colonization rates and strong host specificity. However, translating mechanistic understanding into management prescriptions requires better quantification of the magnitude and persistence of ECM effects on soil C.

### 8.3 Climate change projections

Improving projections of forest C responses to climate change requires better representation of ECM fungi in Earth system models. Key priorities include quantifying how fungal respiration, growth, and enzyme production respond to warming; understanding how drought alters ECM communities and their C cycling functions; determining whether enhanced photosynthesis under elevated CO_2_ leads to sustained increases in C allocation to ECM fungi; and quantifying the net effect of ECM fungi on soil C storage and its feedback to atmospheric CO_2_ concentrations.

## 9 Conclusion

ECM fungi play a central but complex role in forest soil C cycling. Through direct C allocation from host plants, fungal biomass and necromass contribute significantly to soil C inputs. Fungal-mediated soil aggregate formation and organo-mineral interactions provide physical and chemical protection mechanisms that may enhance C persistence. However, ECM fungi also possess enzymatic capabilities for SOM decomposition, and their activity may accelerate C loss through priming effects or decelerate C loss through suppression of saprotrophs (the Gadgil effect).

At the global scale, the context dependency of ECM-mediated carbon cycling is not random but biogeographically structured. The net effect of ECM fungi on soil C storage varies not only with local fungal community composition, host identity, and soil properties, but also with broad-scale gradients in nutrient limitation—from nitrogen-dominated high-latitude systems to phosphorus-dominated tropical forests—and with regionally divergent trajectories of global environmental change. This context dependency explains apparent contradictions in the literature and underscores the need for nuanced understanding rather than generalization.

Key research priorities include: (1) resolving the priming vs. stabilization debate through integrated multi-scale studies; (2) improving representation of ECM fungi in ecosystem and Earth system models; (3) understanding how global change factors alter ECM-C relationships; and (4) translating mechanistic understanding into forest management strategies that enhance soil C sequestration.

Meeting these priorities will require continued methodological innovation, particularly in tracking C fluxes through fungal biomass and linking fungal functional traits to ecosystem C cycling. The importance of forest soils for climate mitigation makes this research essential for informing both scientific understanding and policy decisions.
